# Quartz Crystal Microbalance Method to Measure Nanoparticle–Receptor Interactions and Evaluate Nanoparticle Design Efficiency

**DOI:** 10.1021/jacsau.3c00084

**Published:** 2023-05-12

**Authors:** James A. Behan, Zengchun Xie, Yi-Feng Wang, Xiaoliang Yang, Teodor Aastrup, Yan Yan, Laurent Adumeau, Kenneth A. Dawson

**Affiliations:** † Centre for BioNano Interactions, School of Chemistry, University College Dublin, Belfield, Dublin 4, Ireland; ‡ Attana AB, Greta Arwidssons Väg 21, Stockholm SE-11419, Sweden; § UCD Conway Institute of Biomolecular and Biomedical Research, School of Biomolecular and Biomedical Science, University College Dublin, Belfield, Dublin 4, Ireland

**Keywords:** quartz crystal microbalance, nanoparticles, bio−nano interactions, receptors, interaction kinetics

## Abstract

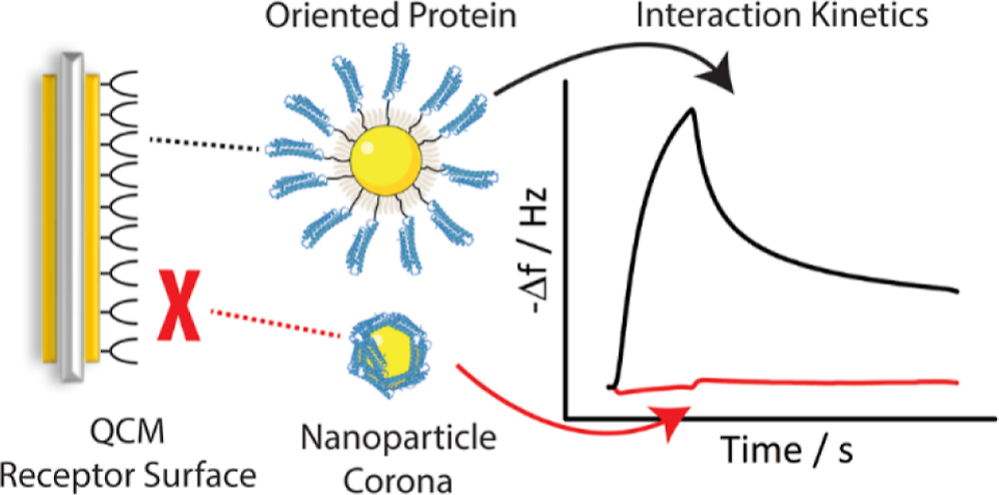

Conjugation of biomolecules on the surface of nanoparticles (NPs) to achieve active targeting is widely investigated within the scientific community. However, while a basic framework of the physicochemical processes underpinning bionanoparticle recognition is now emerging, the precise evaluation of the interactions between engineered NPs and biological targets remains underdeveloped. Here, we show how the adaptation of a method currently used to evaluate molecular ligand–receptor interactions by quartz crystal microbalance (QCM) can be used to obtain concrete insights into interactions between different NP architectures and assemblies of receptors. Using a model bionanoparticle grafted with oriented apolipoprotein E (ApoE) fragments, we examine key aspects of bionanoparticle engineering for effective interactions with target receptors. We show that the QCM technique can be used to rapidly measure construct–receptor interactions across biologically relevant exchange times. We contrast random adsorption of the ligand at the surface of the NPs, resulting in no measurable interaction with target receptors, to grafted oriented constructs, which are strongly recognized even at lower graft densities. The effects of other basic parameters impacting the interaction such as ligand graft density, receptor immobilization density, and linker length were also efficiently evaluated with this technique. Dramatic changes in interaction outcomes with subtle alterations in these parameters highlight the general importance of measuring the interactions between engineered NPs and target receptors ex situ early on in the construct development process for the rational design of bionanoparticles.

## Introduction

Nanoscale biological recognition represents a remarkable assembly of cellular processes in which some nanostructures are (with remarkable fidelity) judged competent to gain access to, and safely transfer information into, the cell against an apparently overwhelming nanoscale background ranging from cellular debris to dust, to pathogens. While the unravelling of these processes is far from complete, the basic conceptual framework is now clear.^1^ In essence, multiple spatially and time-correlated physicochemical interactions at the nanoscale interface between particle surface and cell (outer plasma and internal) membranes trigger peri-membrane cellular processes that capture and transduce sufficient particle information to decide on the nature of the biological response. “Non-permissive” recognition events are often dealt with by default protective mechanisms (trafficking to degradative organelles or activation of broader endogenous defences). Permissive recognition granting access to critical biological machinery (e.g., RNA metabolism in cytoplasm) is a gated process based on specific architectural cues, typically in a narrowly defined window of binding energy and exchange times with specific groups of receptors and transmembrane protein clusters. Our capacity to develop and apply nanostructures in biology and medicine (from cell–pathogen interactions to nanoscale medicines) is crucially dependent on understanding these collective nanoscale physicochemical interactions at the interface, mastery of the peri-membrane information capture and transduction processes, and, crucially, the interconnection between these. In the hands of chemists, the science of synthetic engineered bioconjugation has developed to a significant degree, but it could advance much further, given sufficient clarity on what constitutes “permissive” biological interactions and easy access to efficient tools to assess those nanoscale interactions. Here, we will focus on one aspect of that problem: the measurement of interactions, exchange times, and the relationship to simple current particle design parameters.

Interactions between nanoparticles (NPs) and receptors have been extensively investigated on cells.^2−4^ This often involves knock-in or knock-out gene expression to validate the specificity of the receptor recognition. While such approaches provide information on key molecules for bio–nano interaction, it does not advance our understanding on the interface engagement between NPs and receptors. Currently, very few bionanostructures (synthetically or endogenously derived) are subjected to measurement of interactions, and this leaves us without a real developmental pathway connecting structure to function.^5^

Here, we present a quartz crystal microbalance (QCM)-based method to directly measure kinetic stages of the NP and receptor engagement to uncover the roles of several particle design parameters in particle targeting. The QCM technique uses shifts in the piezoelectric oscillation frequency of quartz crystals to detect extremely small changes in adsorbed mass per unit area, typically sensitive to nanograms per cm^2^.^6^ One of the key advantages of the QCM approach is its versatility: the technique has been applied in studies of biological interfaces, including antibody–antigen interactions and can be applied in virus detection and biosensing. When coupled with dissipation monitoring (QCM-D), changes in the viscoelastic properties of the interface, for instance conformational changes in lipid vesicles, bilayers, or grafted proteins, can be measured.^7−9^

The QCM can also be integrated with other techniques applied in surface interaction studies such as surface plasmon resonance, localized surface plasmon resonance,^10−12^ and spectroscopic ellipsometry,^13^ which also affords complementary information on changes in the mass, thickness, and composition of the biological layer.

By immobilizing on the QCM surface one of the biomolecule partners from a receptor–ligand couple, it is possible to measure the interactions between the two molecules by following the change of the oscillation frequency over time. As the interactions can be recorded over time, it is possible to collect kinetic information about the interaction. However, when kinetics are recorded, the effect of mass transport limitations should be considered. This well-known effect in measuring biomolecular interaction kinetics,^14^ which has a potential impact in vivo,^15^ becomes even more significant for NPs due to their larger sizes and lower diffusion coefficients. To further compound the situation, while reducing the mass transport limitation (by increasing the flow rate or reducing the density of immobilized receptors) produces results that are more representative of the actual interaction kinetics, this is not always possible in practice and can itself lead to loss of information in the measurements. Alternatively, we argue that we can focus on comparative measurements in which particle size, shape, and target surface are fixed, and while mass transport contributions remain, one can still learn much about the interactions and qualify the outcomes of different synthetic strategies. Dissipation monitoring was not relevant for this study because the potential receptor–ligand conformation changes would be insignificant compared to the overall scale of the large solid NPs used here. Additionally, because the mass of each NP is not expected to change during the interaction, the frequency shift is sufficient on its own as a measure of the number of NPs interacting with the surface.

Apolipoprotein E (ApoE) was chosen as an example of a targeting biomolecule conjugated on the NPs because ApoE and its fragments that bind to the low-density lipoprotein receptor (LDLR) and other lipoprotein receptors have been used to functionalize a diversity of nanostructures to cross the blood–brain barrier.^16−19^ On the other hand, most nanostructures (including surface-functionalized structures) are well recognized in vivo by the mononuclear phagocyte system (MPS), which expresses abundant scavenger receptors such as the macrophage receptor with a collagenous structure (MARCO),^2,20^ scavenger receptor B, and others.^21,22^ It has long been a concern that complex surface arrangements involving the biomolecular corona and targeting moieties may lead to such undesired interactions. Therefore, in this study, we generated a set of ApoE-functionalized NPs with systematically varied design parameters, including degree of targeting moiety orientation, average grafting density, and choice of linker (and co-linker) chemistry and lengths, and evaluated the kinetics of interaction with both “on-target” ApoER2 and LDLR and with the scavenger receptor MARCO. We demonstrate that QCM can be a powerful technique to evaluate the interactions between specific receptors and different bionanoconstructs and to identify the important design parameters, allowing for optimal recognition.

## Results and Discussion

### Design of ApoE-Functionalized NPs and QCM Measurement for the Study of NP–Receptor Interactions

**Figure 1:**
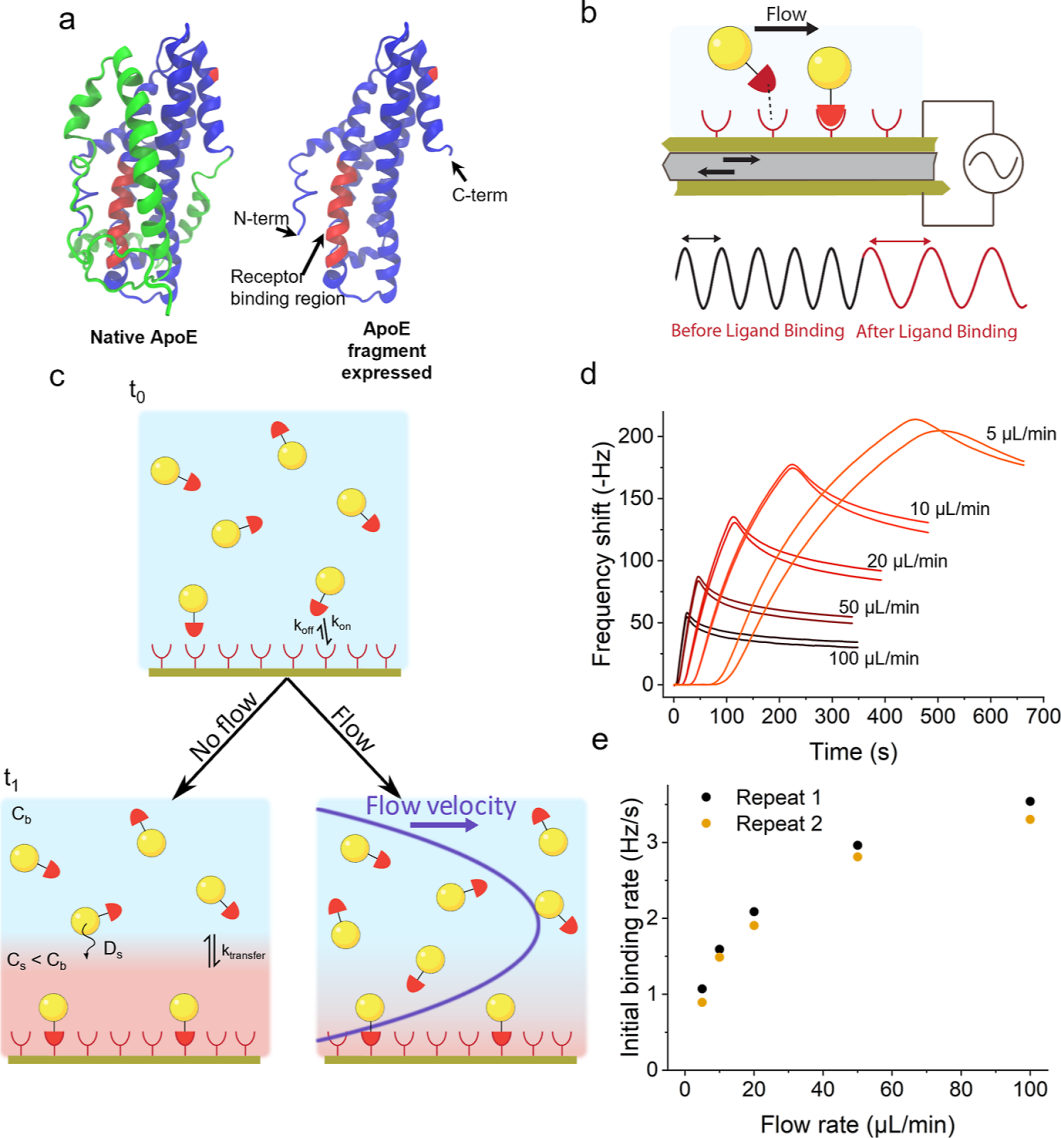
(a) Structure of the native ApoE and the expressed fragment. In the native ApoE, the protein C-term domain (in green), involved in interactions with the lipids, blocks direct access to the receptor binding region (in red). Hence, in the expressed fragment, the C-terminal domain has been truncated (PDB ID 2L7B). (b) Principle of QCM: dispersion of NPs interacting with the receptor immobilized on the quartz crystal surface increases the mass of the vibrating crystal and affects the frequency of oscillation. Recording the change of this frequency over time gives an indication of the variation of mass adsorbed at its surface. (c) Representation of the mass transport limitation: formation of a depletion layer in the case where the diffusion of the NPs to the surface is slower than their association to the receptor. The concentration of free NPs in this depletion layer is inferior to the bulk concentration, leading to a reduction of the apparent association rate constant. A lateral flow reduces the impact of the mass transport limitation by limiting the extent of the depletion layer. (d, e) Impact of the lateral flow rate setting on the apparent initial association rate, indicating the effect of the mass transport limitation.

To allow oriented grafting of ligands at the surface of SiO_2_ NPs, we designed and expressed a recombinant ApoE fragment corresponding to the 22 kDa N-terminal structural domain including the LDLR-binding site,^17,23^ engineered to possess only a single cysteine at either the C- or N-terminus (respectively named ApoE-CT and ApoE-NT) (Figure 1a and Supporting Information). The single cysteine, in either the N- or C-terminus, was then used to conjugate the protein onto the NP’s surface, thus allowing the control of their orientation. The conjugation between the protein and NPs was done using maleimide–sulfhydryl chemistry and heterobifunctional poly(ethylene glycol) (PEG) linkers.^48^

The principle of the measurement consists in coating the sensor surface with a variety of relevant target receptors for which the functional receptor recognition domains may be isolated (Figure 1b). The binding of NPs leads to an increase in the mass of the vibrating crystal, which in turn affects the frequency of oscillation. As represented in Figure 1c, and introduced earlier, when the particle dispersion contacts the surface of the sensor, rapid initial particle binding leads to a depletion layer (in red) and if diffusion to the surface (*D*_s_) (and particle transfer across the depletion layer to the surface, *k*_transfer_) is slow compared to the association rate, then the QCM kinetic constants are mass transport-limited. A lateral flow of the carrier fluid across the surface reduces the extent of the depletion layer, potentially allowing us to measure intrinsic particle–surface binding kinetics with minimal mass transport effects. The initial binding rates may be used to approximate the particle–surface association kinetic rate because the dissociation contribution may be neglected during the initial phase. With increasing flow rate, measurements of the binding kinetics between ApoE-functionalized NPs and ApoE R2 (Figure 1d,e) evolve asymptotically toward the non-mass transport-limited association rate, and it becomes meaningful to cite an effective rate constant that largely characterizes the particle interaction with the target rather than the experimental set-up. However, practically, in QCM, the useful range of flow rates is limited by the requirement for sufficiently long recording times in order to obtain an accurate estimate of the association kinetics and by the volume of sample required, which may be limiting in the case of highly specialized constructs. Such limitations could in future be reduced by technological developments consisting, for example, in cycling the sample or using an alternating flow; here, unless otherwise specified, we restrict the discussion to experiments with a single flow rate (10 μL/min), which for our system gave a good compromise between the aforementioned constraints of mass transport, association kinetics, and sample volumes.

Given the important role of receptor (and other membrane protein) clustering in NP cell interactions,^24−28^ clearly prescribed reproducible spatial organization and orientation of the receptors at the surface would be more representative of true membrane recognition, but new sensor developments will be required to address those questions. Also, while we can reproducibly control the density of the immobilized receptor during the functionalization of the sensor by following its frequency change, it is more precise to regenerate a single prepared sensor surface between the measurements by inducing the release of bound ligands. Assuming an efficient regeneration, this allows for a direct comparison of different NP constructs against a single reference surface, and hence care was taken here in optimizing methods, protocols, and conditions to allow for effective regeneration (Supporting Information, Figure S12). Regardless, successive cycles of regeneration inevitably lead to the inactivation of receptors; to account for this, we have reported duplicate measurements of each experiment throughout.

### Role of Ligand Orientation and Graft Density in Interactions and Exchange Times

**Figure 2:**
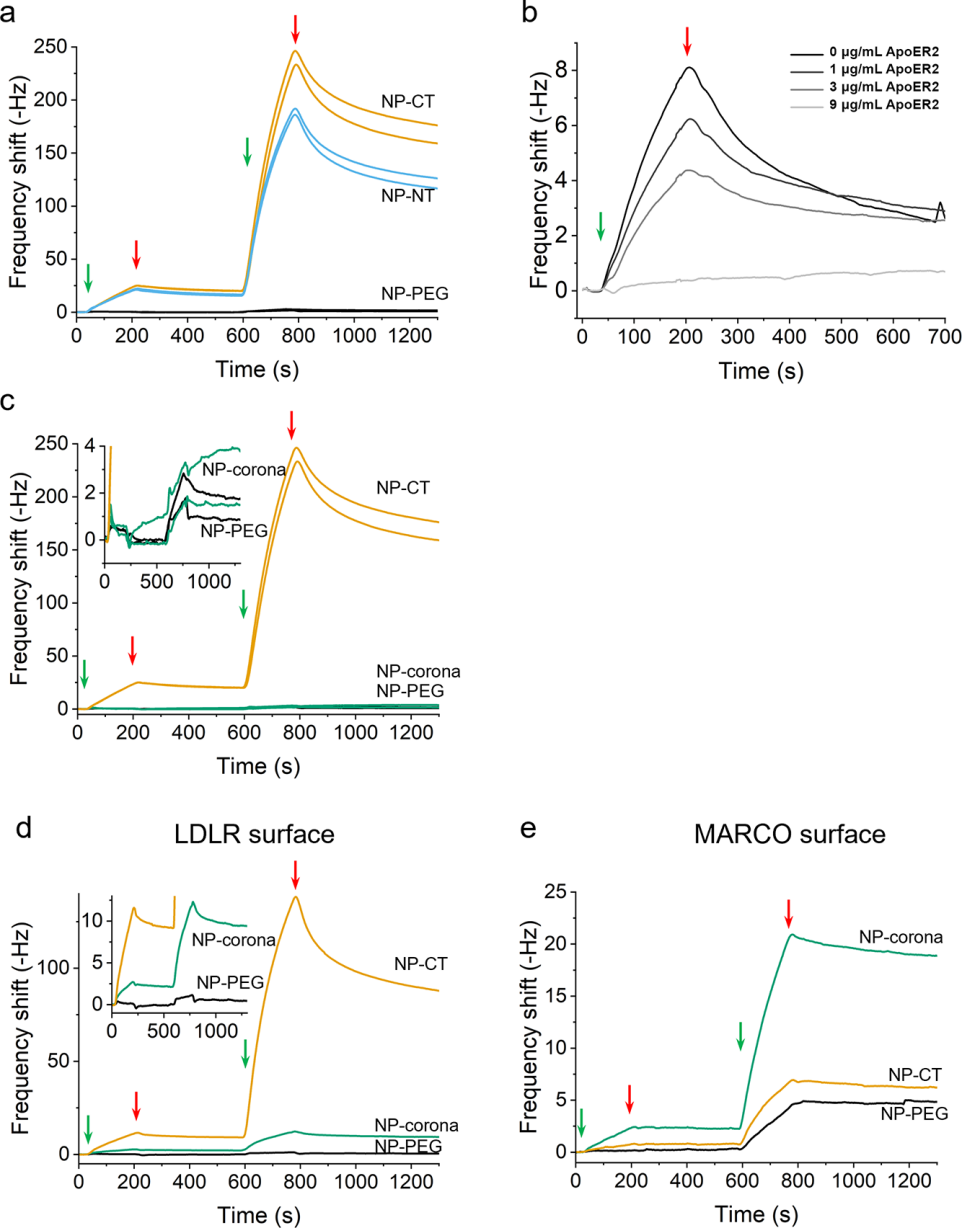
(a) Traces comparing the binding to ApoE R2 of the two different protein orientations grafted on the NPs (NP-CT and NP-NT with about 400 proteins/NPs). Constructs were injected at 20 and 200 μg/mL (injection happening between the green and red arrows) without regeneration of the surface between the 2 injections. The results are shown in duplicate. (b) Investigation of specificity of NP-ApoE construct interactions with ApoER2 surfaces. Constructs at a concentration of 20 μg/mL were pre-mixed with free ApoE R2 at increasing concentrations and injected onto an immobilized ApoER2. Total suppression of interaction was achieved with 9 μg/mL of free receptor. (c–e) Binding profiles obtained for NPs with grafted (NP-CT) and adsorbed ApoE (NP-corona) on immobilized ApoE R2 (∼20 Hz) (c), LDLR (∼60 Hz) (d), or MARCO (∼40 Hz) (e). Constructs were injected at 20 and 200 μg/mL (injection happening between the green and red arrow) without regeneration of the surface between the 2 injections.

We begin by illustrating the differences in interactions between individual (interaction domain) ligand–receptor interactions and when multiple copies are organized differently, starting with the example receptor binding fragments of ApoE (ApoE-CT, ApoE-NT). Circular dichroism spectra of the two binding fragments suggest that the secondary structure is comparable to that within the native ApoE (Supporting Information, Figure S1), and alterations in amino acid sequence to facilitate oriented bioconjugation did not greatly disturb the native secondary structure. It has been suggested that (in the absence of associated lipids^23,29^) the binding of ApoE to ApoER2 and related receptors is weak, and indeed, we found that isolated ApoE-CT and ApoE-NT fragments appear to interact weakly with the ApoER2 surface unless associated with small unilamellar vesicles of the phospholipid DOPC (Figure S2). In order to obtain functionalized NPs with the two different protein orientations, ApoE-CT and ApoE-NT fragments were grafted on SiO_2_ NPs using a short poly(ethylene glycol) (PEG) composed of 8 ethylene glycol units (Xie et al., unpublished). The resulting constructs with C-terminal or N-terminal fragments are termed NP-CT and NP-NT, respectively. The use of a short PEG linker in this case was motivated by the idea of limiting the mobility of the grafted protein in order to maintain a favorable orientation for receptor binding. In Figure 2a, we compare typical QCM binding curves of NP-CT and NP-NT, each having ∼400 proteins per NP (protein amounts determined via the BCA assay, Figures S5–S7), on the immobilized ApoE R2 fragment (immobilization corresponding to a shift of 16 Hz). The control construct NP-PEG (NPs identical to those used to prepare NP-CT and NP-NT but with no grafted protein) shows minimal binding, while both the C- and N-terminus constructs show a clear binding to the ApoE R2-functionalized surface. While the difference is small (and could be attributed to small differences in ligand density), binding of NP-CT appears to be stronger than for NP-NT, suggesting that the fragment immobilized in the C-terminal orientation is better presented to the receptor binding motif. To demonstrate the binding specificity, NP-CT (at 20 mg/L) was pre-incubated with increasing concentrations of free ApoE R2 receptor recognition domain fragments before injection on the ApoE R2-functionalized surface were investigated. Figure 2b shows the result of this competition experiment, demonstrating that the interaction between the grafted NPs and the receptor surface diminished with increasing concentrations of the free receptor, suggesting that we are indeed measuring specific surface bound ligand–receptor interactions. On the other hand, adsorption (rather than oriented grafting) of the ApoE fragment (“non-specific NP-corona”) fails to generate receptor interactions (Figure 2c) even at very high densities of the adsorbed protein on the NPs (10^3^ proteins/NP, Figure S4) on the same batch of NPs used for the oriented protein grafting. Considering that these densities of the adsorbed protein are significantly higher than those obtained for the oriented NP-CT and NP-NT constructs, this result highlights the need for a meaningful evaluation of NP–receptor interactions ex situ prior to prospective in vivo NP targeting studies.

To demonstrate the broad applicability of our approach, we extended the study to the LDLR (which also recognizes ApoE) and compared the interactions of NP-CT and NP-NT constructs to those measured with ApoE R2. In Figure 2d, it is clear that the grafted construct NP-CT is strongly recognized by the LDLR surface, and the NP–Corona–LDLR interaction is significantly weaker than for the grafted construct despite the fact that the number of proteins in the corona is much larger than in the grafted layer (Figure S4). This result agrees with the literature, showing that adsorbed ApoE bionanoparticles, while showing recognition by LDLR and other receptors in vitro, interact less efficiently.^30^ Moreover, these simple architectures based on adsorbed proteins may also be subject to more severe off-target effects. For instance, scavenger receptors underlie many of the key clearance mechanisms in vivo (for example, liver clearance) and are known to recognize NP-corona constructs in the biological milieu.^2^ In many cases, this recognition is likely to be linked to nonspecific corona protein adsorption from the milieu, but the precise mechanism of this interaction in the absence of such effects is poorly understood and could involve de novo recognition motifs present in adsorbed or poorly grafted ligands. Figure 2e shows binding curves obtained for the constructs with random adsorption (NP-corona) or oriented ligand (NP-CT) with MARCO. While oriented NP-CT constructs show no significant interaction with MARCO compared to NP-PEG, the NP-corona is clearly strongly recognized by MARCO. This is an example where, quite independent of the role of non-specific adsorption (long believed to be significant), poorly organized ligands on nanostructures may not only result in a weak interaction with the target receptor but also create off-target effects (including recognition by scavengers and subsequent clearance by MPS).

### Role of Ligand and Receptor Immobilization Density

**Figure 3:**
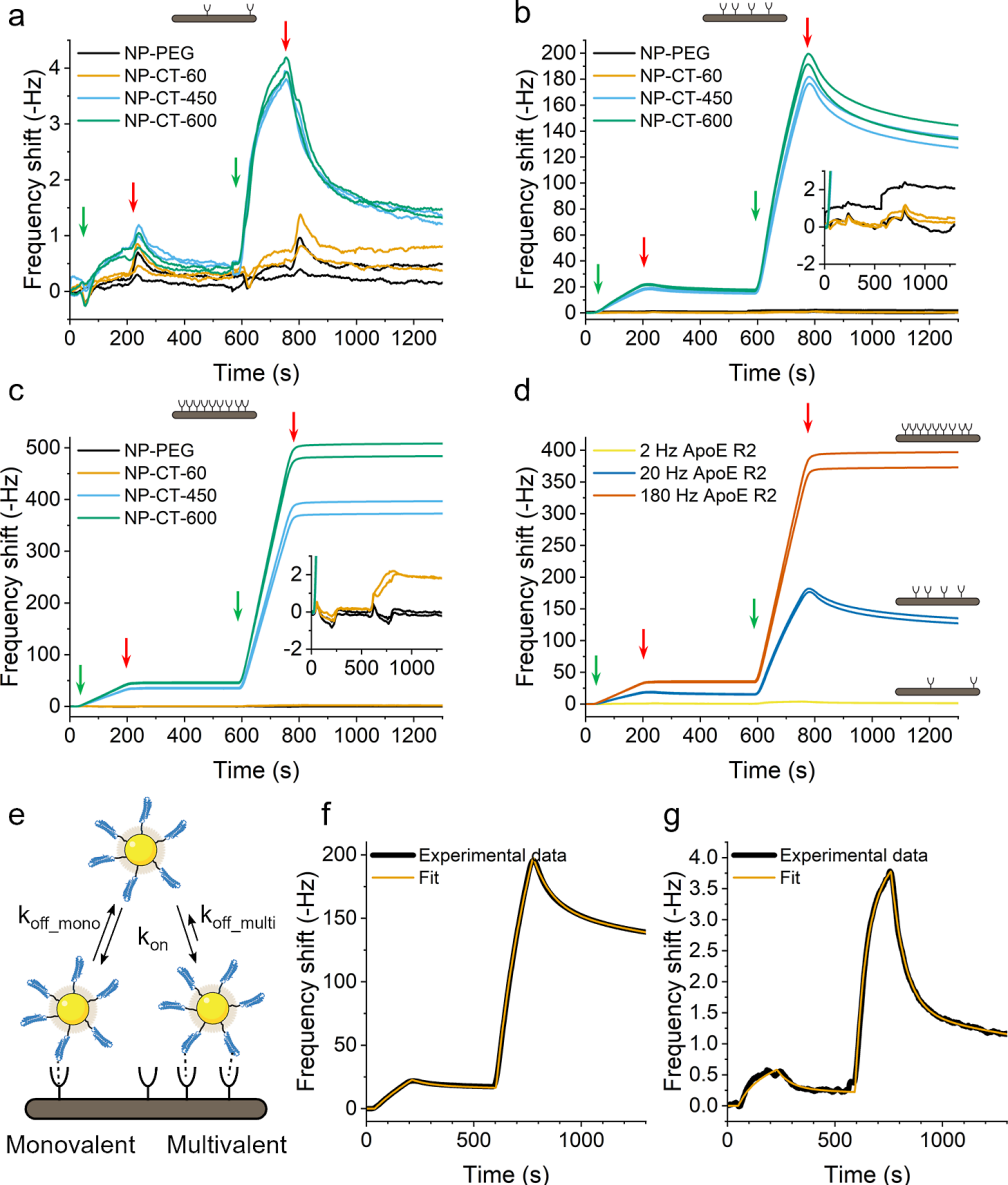
(a–c) Study of the interaction profile between NP-CT with different grafting densities (number of proteins per NP indicated in the name) and different receptor densities (respectively 2, 20, and 180 Hz). (d) Superimposition of the graph obtained with NP-CT-450 for the 3 different receptor densities. Green arrows indicate injection of 20 mg/L (left) and 200 mg/L of NPs (right). Red arrows indicate the end of the injection. Repeats of the experiment after surface regeneration are presented to demonstrate reproducibility. (e) Multivalent model of binding of NP-CT constructs to ApoER2 surfaces. (f, g) Fits with the model in (e) of NP-CT-600 on medium and low receptor densities of ApoER2, respectively.

We next investigated the particle–receptor (ApoE R2) interaction at a range of different protein graft densities and receptor immobilization densities (2, 20, and 180 Hz corresponding to approximate average receptor separations of ca. 44, 14, and 5 nm, details of the calculations in the Supporting Information, Table S1). The results reported for C-terminal-grafted ApoE fragments in Figure 3a–c show little evidence of interaction for the construct NP-CT-L with low densities (ca. 60 proteins/NP) compared to the control NP-PEG constructs even at the highest receptor densities (∼180 Hz) (Figure 3c) where the surface is estimated to be saturated with receptors (Supporting Information, Figure S11). When the grafting density is increased to ∼400 proteins/NP, the NP-CT constructs measurably interact with ApoER2 even in the case of the lowest receptor density (Figure 3a). ApoER2 is a complex receptor with a repetition of the binding unit,^31,32^ and it is possible that in order to measure significant binding, several ApoE motifs may need to interact with the same receptor.

**Figure 4:**
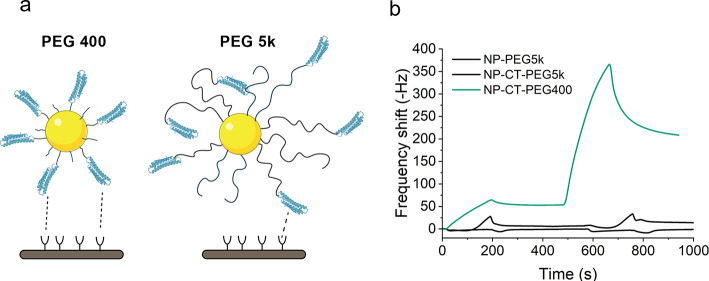
(a) Illustration of NP-CT with PEG-400 and PEG-5k linkers. Shorter PEG linkers present a lower steric hindrance to binding and increase the probability of preserving the orientation of the grafted protein, while longer PEG linkers are bulkier and allow for the “burying” of binding domains due to their flexibility. (b) Comparison of association profiles between NP-CT-PEG400 and NP-CT-PEG5k obtained by QCM on a surface with ∼20 Hz of ApoER2. Injections of 20 and 200 μg/mL NPs were used in each case; no binding was observed for NP-CT-PEG5k.

To facilitate this, grafted proteins must be close enough one another at the NP surface to simultaneously interact with a single receptor. The probability for this event to happen is proportional to the protein density at the surface of the NPs, which immediately suggests that a “threshold” of adjacent proteins in a suitable orientation should be reached in order to observe binding. However, we stress that even for oriented protein grafting, the distribution of grafted proteins on the surface of the particles is uncontrolled in terms of surface arrangement, and the exact mechanisms at play remain unclear.

The ApoE-ApoER2 system illustrates the utility of the QCM screening approach for characterizing engineered nanoconstruct functionality. When evaluating engineered bionanoparticles in vitro or in vivo, there is a tendency to evaluate their success or failure according to their uptake or accumulation. However, we have shown that a system consisting of only a single protein/receptor pairing may fail to yield any quantitative interaction even at unrealistically high receptor densities and that subtle changes (here in orientation and density) yield the opposite result. Hence, it is premature to declare certain targeting approaches as viable or not based on existing in vivo studies without first evaluating these interactions.

A further point of note from Figure 3a is that the association and dissociation curves for NP-CT remain unchanged even when the protein grafting density is increased to more than 600 proteins/NP, suggesting that, for low ApoE R2 densities, the predominant interaction is between ligands on the NP and a single immobilized receptor. This suggestion is also supported by the estimated average distance between the receptors of ∼44 nm, leading to a small probability for the constructs to interact with multiple receptors. In contrast, for medium and high receptor density, the dissociation phases are drastically different than the one observed for the low receptor density. We associate this observation to the possibility for the multivalent NPs to interact with several receptors at the same time. The QCM only detects the binding of a construct to the sensor surface and does not distinguish between individual or multivalent receptor–ligand interactions. Hence, an increasing number of binding pairs *n* leads to a reduction of the equilibrium constant *K* according to the equation , where Δ*G*° is the binding free energy of a single receptor–ligand interaction, *R* is the gas constant, and *T* corresponds to the temperature. When *n*·Δ*G*° ≫ *RT*, the interaction equilibrium is strongly shifted toward the associated state, and the binding appears irreversible. In other words, in the case of multivalent interactions, the construct dissociates from the surface only when all the individual interactions have dissociated. As the probability for this combination of events is far lower than in the case of a monovalent interaction, the apparent dissociation rate is also slower than that observed for monovalent particles. This conclusion is consistent with estimates of average receptor–receptor distances of ∼15 and ∼5 nm for the medium and high density, respectively (Table S1), allowing the particles to interact with several receptors at the same time. It is important to recognize that in targeting applications, contrary to expectations, strong irreversible binding may well not be the desired outcome for permissive bionanoscale recognition. Indeed, our current understanding suggests that combinations of inappropriate spatially coordinated ligands and exchange times that exceed tightly defined windows both lead to non-permissive recognition events and in some contexts represent “danger signals”.^1^ When the interactions of sufficiently well-designed particles are studied, it becomes possible to rationally choose regimes of exchange times within those acceptable limits.

The density-dependent binding behavior observed in Figure 3 suggests the presence of multivalent interactions. Multivalency is complex to model,^33,34^ considering, for example, that the kinetic parameters for the first interaction would potentially be different from the following interactions due to entropic reasons. Moreover, the binding between ApoE and ApoER2, or other receptors from the LDL receptor family, is complex due to the repetition of the binding domains on the receptor and potential conformational changes induced by the binding.^31,32^ In addition to these factors, one must also consider the surface heterogeneity on the QCM sensor as well as the heterogeneity existing in the population of NPs and the mass transport limitation. Sufficiently elaborated models have not yet been developed for the NP regime described here despite the attempts to develop models describing the complexity of multivalency.^35−38^ Moreover, complex models come with an increased number of parameters, leading to a better fit but also to lower confidence in the fitted values due to correlation between variables.^39−41^ As such, it would not be appropriate to overinterpret models trying to describe multivalency, but it is still of some interest to fit the kinetic curves (using ClampXP, see the Supporting Information) in order to qualitatively evaluate the role of such interactions. For that purpose, here, we consider a simple heterogenous surface model with two different sites: one “monovalent” site with a specific association and dissociation constant and one “multivalent” site with the same association constant but a different dissociation constant (Figure 3e). From this model, we derive an apparent association constant (*k*_on_), the apparent dissociation constants for the 2 sites (*k*_off_mono_, *k*_off_multi_), and the relative number of multivalent sites compared to monovalent sites. Example fits using this model in Figure 3f,g show that the model gives an excellent fit of the experimental data (NP-CT-600 on medium (20 Hz) and low (2 Hz) receptor density, respectively) (see Table S2 for the full details). Interestingly, the result of the fits indicates that the apparent fraction of “multivalent” sites increased with increasing receptor density. As shown above, the increase of the receptor density also increases the probability for multiple receptors to be in close proximity to one another to facilitate multivalent interactions.^36^ Apparent association rate constants are of the order of 10^7^ M^–1^ s^–1^ for both low and medium density receptor surfaces, while the two surface densities have a dissociation constant of the order of 10^–3^ s^–1^ for “monovalent” sites and 10^–4^ s^–1^ for “multivalent” sites. Such an interpretation is also consistent with the observation that, for yet higher receptor surface densities, particles with sufficient graft density to support multivalent binding become essentially irreversibly bound (on the timescale of the experiment). The apparent affinity constant *K*_D_ calculated for the monovalent site would be of the order of 10^–10^ M. The precise *K*_D_ for the interaction between ApoE and ApoER2 appears to be unknown due to the complexity of the system itself; however, Brandes et al. reported an affinity between ApoER2 and apoE-rich β-VLDL of the order of 10^–8^ M.^42^ In the case of the ApoE–LDLR couple, an affinity of the order of 10^–10^ M has been reported.^43^ These values are broadly consistent with the approximate *K*_D_ we report here and in a biologically relevant range.

### Effect of PEG Linker Length on Bionanoparticle–Receptor Interactions

We next investigated the effects of linker choice, linker length, and graft density (of both linker and ligand) on receptor steric access. We therefore repeated the medium-density immobilization on the NPs using varying lengths for the PEG: the one previously used of 400 g/mol and a PEG of 5 kg/mol (grafted NPs, respectively, named NP-CT-PEG400 and NP-CT-PEG5k).

It was observed (Figure 4) that, even if the protein density between the particles with the two different PEG lengths was comparable (as determined by BCA assay), NP-CT-PEG5k did not interact with the medium density receptor surface, whereas the NP-CT-PEG400 does interact with the same receptor surface, as already seen in Figures 2 and 3. Additionally, we verified that when using a PEG of 1 kg/mol and comparable protein grafting densities, NPs exhibited strong ApoER2 recognition (Figure S14). This suggests that excessively long linkers may hinder access of the ligands to receptors even at relatively high graft densities. As a minor remark, we also note that the fluorescence emission spectra of solutions containing NP-CT (PEG400 and PEG5k) with identical protein concentrations exhibit a fingerprint (*l*_max_ close to 330 nm) similar to lipid-bound ApoE (Figure S15),^17^ which was already shown to be strongly recognized by the receptor ApoER2 (Figure S2). This suggests that the lack of NP–receptor interactions observed with the longer linker is not due to unfavorable changes in grafted protein conformation. Also, while it is not possible to make direct comparisons to cell interactions, Maslanka Figueroa et al. observed a reduction of cell uptake in studies of multivalent NPs prepared having 5 kDa PEG linkers^44^ compared to mixed 2 and 5 kDa linkers. Such observations have been attributed mostly to the inhibition of multivalent interactions with longer linkers due to an increase in the lateral steric hindrance. However, it is also possible that this observation could be the result of the “end effects”, well known from polymer physics, in which the chain termini begin to internalize and could lead to a reduced accessibility of the grafted ApoE recognition domain.

Regardless of the reason, we stress again that our study of the impact of linker effects on the binding was accomplished using a single reference surface of receptors in order to facilitate a meaningful and quantitative comparison between different bionanoparticles. When the technique is complemented by other standard techniques (such as spectroscopic characterization of the protein conformation, as we argued), it is possible to attribute the results to some feature of the construct design with confidence. After refinement of the design (here, the choice of linker, previously the choice of orientation and density), the interaction can be quickly reevaluated ex situ and under exchange times of broad biological relevance.

## Conclusions

Bionanoscale recognition should be more broadly seen as a complex, integrated series of interaction and other perimembrane events involving particular biomolecular motifs at the interface between the NP and cell surface. While many of these steps are defined also by downstream processes stimulated by spatial nanostructure surface architecture (and related organizational elements not solely related to initial interactions), the facts remain that the strength and lifetimes of the physical interactions remain a key part of the initial information on nanoscale identity collected by the cell. Rational developments of functional bionanostructures requires the alignment of particle synthetic chemists and engineers to qualitatively and quantitatively explore those interactions using cell-free methods that make a transparent connection between structure and interactions. We argue that the field of NP synthesis and grafting has advanced sufficiently to deliver much more if these interaction studies are brought into focus. Indeed, we show that commonly applied bionanoscience approaches that confer biological identity using (for example, randomly or other) attachment methods for the recognition motifs to particles may fail to meaningfully engage with the biological target. Also, reported failures, for example, in vivo, may therefore not be conclusive.

We should caution that all our evidence from bionanoscale recognition does indeed suggest that we do need to go beyond current ideas of targeting. However, in the broader interests of scholarship, before drawing too broad conclusions that certain approaches to targeting “do not work”, it would be wise to recognize that, without knowing such information about the interactions, they have not been fully evaluated. Whatever the outcome here, our opinion is that only by making such measurements can we isolate and rank the various contributions that lead to off-target (or target failure) effects and plot a rational path forward.

## Experimental Section

### Chemicals and Materials

Hydrochloric acid (37%), sodium dodecyl sulfate (98.5%), glycerol (>99%), 1,4-dithiothreitol (>98%), bromophenol blue, tetraethyl orthosilicate (98%), fluorescein isothiocyanate isomer (>90%), 3-aminopropyltrimethoxysilane (>97%), ammonia (35%), ethanol (99.8%), acetic acid (analytical standard), 2-mercaptoethanol (>99%), (4-(2-hydroxyethyl)-1-piperazineethanesulfonic acid (HEPES, 99.5%), glycine (>99%), succinic anhydride (>99%), *N*-(3-dimethylaminopropyl)-*N*′-ethylcarbodiimide hydrochloride (EDC), *N*-hydroxysulfosuccinimide sodium salt (>98%), NaCl (>99.5%), Tween 20, and dimethylsulfoxide (>99.9%) were purchased from Sigma-Aldrich and used without further purification. Multicolor broad range protein ladder, Micro BCA assay kit and Pierce LAL chromogenic endotoxin quantitation kit were purchased from Thermo Fisher Scientific. NHS-PEG8-Maleimide was purchased from Irish Biotech GMBH. Normal Human serum was purchased from Merck-Millipore Inc.

#### Proteins and Receptors

Bovine serum albumin (>96%) was purchased from Sigma-Aldrich. Recombinant human MARCO protein (>95%) was purchased from R&D Systems. The recombinant human low-density lipoprotein receptor and ApoE receptor 2 (>90%) were purchased from Neuromics Inc. Recombinant human ApoE3 was purchased from Peprotech Inc.

### Nanoparticles

PVC microspheres (0.263 μm) were purchased from CPS Instruments Inc. for use as a standard in DCS experiments.

#### NP-Corona Formation

NPs at a mass concentration of 1 g/L were incubated in the solutions of the protein in HEPES 50 mM at an indicated concentration for 1 h at 37 °C. After establishment of the adsorption isotherm, corona formation was carried out at a concentration of 1 g/L ApoE for the NPs used in the QCM. Following incubation, hard protein corona complexes were obtained by centrifugation of NPs at 4 °C to pellet the particle–protein complexes separately from free proteins. Following this, resuspension and washing steps with 10 mM HBS were used to remove low-affinity proteins and the final NP-corona pellet was resuspended in the working buffer for the experiment.

#### Synthesis of ApoE-Grafted SiO_2_@FitC NPs

The ApoE-Grafted SiO_2_@FitC NPs were prepared according to a protocol developed within the group by Xie et al. (unpublished), which was carried out under sterile conditions using certified endotoxin-free plastic wares and reagents. All glassware used was cleaned and sterilized using either Aqua Regia (in the case of flasks, connectors, and stir bars) or by being heated at 200 °C in an oven for 2 h before being transferred to a previously sterilized biological hood prior to use. All manipulations of the particles at each stage were performed under biological hood following an aseptic technique. Particles were confirmed to be free from endotoxin contamination using commercial chromogenic endotoxin quantitation kits (Figure S3).

## Methods

### NP Characterization

#### Dynamic Light Scattering

Dynamic light scattering (DLS) measurements and zeta potential determination were carried out using a Malvern Zetasizer equipped with a back-scattering (θ = 173°) detector. Each measurement was an average of three measurements. Cuvettes and zeta potential cells were pre-equilibrated to 25 °C in the instrument for 2 min prior to the start of the measurement. Data analysis was carried out using the Zetasizer software using a cumulant expansion of the field autocorrelation function to the second order. Moreover, in order to obtain the particle size distribution, a constrained regularization method, Contin, was used to invert the experimental data.

#### Differential Centrifugal Sedimentation

Differential centrifugal sedimentation (DCS) measurements were carried out using a CPS Disc Centrifuge DC24000 as reported previously.^2,45,46^ Briefly, the optically transparent centrifuge disc was filled with a sucrose density gradient of fixed composition in order to stabilize particle sedimentation against streaming. This gradient composition is protected against evaporation by the addition of a dodecane layer. The particle size (relative number %) was determined by injection of the sample at a concentration of *ca.* 1 mg/mL at a disc rotation speed of 18,000 rpm. Each measurement was first calibrated using a PVC standard of diameter 263 nm. The size distribution is determined via measuring the time taken for particles to sediment from the injection point at the center of the disc through the gradient to a detector placed at the outer rim of the disc via turbidity measurements. The resulting data are converted by the software (CPS Instruments) to size distributions by particle number. For protein-coated systems such as the NP-corona, the resulting distributions are calculated assuming a spherical shape and uniform material density but could be corrected to account for differences in the protein shell density from the NP density using previously reported models.^47^

#### Transmission Electron Microscopy

Transmission electron microscopy (TEM) images were obtained using an FEI Tecnai G2 20 Twin transmission electron microscope using an accelerating voltage of 200 kV. Samples were prepared by evaporating ca. 5 μL of a 0.5 mg/mL particle suspension onto formvar-coated copper grids (Agar Scientific), 400 mesh.

#### Fluorescence Spectroscopy

Fluorescence measurements were carried out using a Horiba Fluorolog. A quartz cuvette (Hellma Analytic) with a path length of 10 mm was used. Fluorescent SiO_2_@FitC NPs were excited at a wavelength of 488 nm, and emission spectra were measured between 500 and 600 nm. A slit size of 5 nm was used for both excitation and emission along with an integration time of 0.1 s across 1 nm intervals. Background fluorescence spectra in ultrapure water were subtracted from the measured spectra of the NPs prior to reporting.

#### Sodium Dodecyl Sulfate-Polyacrylamide Gel Electrophoresis

Samples were dissolved or suspended in home-made loading buffer (62.5 mM Tris–HCl pH 6.8, 2% (w/v) SDS, 10% glycerol, 0.04 M DTT, and 0.01% (w/v) bromophenol blue) and boiled for 5 min at 90 °C. Samples of equal volume were loaded in 10% polyacrylamide gels. Gel electrophoresis was performed at a constant voltage of 120 V. Afterward, gels were stained for 1 h in Coomassie blue staining (50% methanol, 10% acetic acid, 2.5% (w/v) brilliant blue) and destained overnight in 50% methanol and 10% acetic acid solution. Gels were imaged using a Biorad GS-800 calibrated densitometer scanner, and resulting images were analyzed and exported using commercial GeneSYS software.

### Quartz Crystal Microbalance

#### Surface Immobilization of Receptors and Proteins

QCM measurements were carried out on a dual-channel Attana Cell 200 instrument. In a typical experiment, Attana LNB-Carboxyl QCM chips were functionalized with proteins according to a standard EDC-sNHS coupling procedure. Briefly, new chips were inserted into both channels and equilibrated in HBS-T buffer until a baseline drift <0.5 Hz/min was achieved. At this point, the carboxylated surface of both chips was activated using a 50 μL injection of 200 mM EDC and 50 mM sNHS at a flow rate of 10 μL/min. Afterward, the receptor of interest was immobilized to the desired density on the experimental (“A”) channel only by diluting the stock in acetic acid buffer 100 mM, pH 4.5, and injecting at a flow rate of 10 to 50 μL/min (depending on the required receptor density). After this immobilization, the A channel and the control (“B”) channel were blocked through serial injections of BSA (50 μg/mL in acetic buffer) until saturation was observed, and finally, the remaining activated carboxyl moieties were blocked using a solution of 1 M ethanolamine, pH 8.5.

### Investigation of Receptor Interactions of ApoE-SiO_2_@FitC and Other Bionanoparticles

After surface immobilization of the target receptor as described above, the running buffer was changed to a 50 mM HBS buffer with 1 mM CaCl_2_ and 0.1% BSA, unless otherwise specified. Interaction studies were carried out by injecting 30 μL of the desired analyte dissolved or suspended in a solution of identical running buffer into both A and B channels at varying concentrations (protein or NPs with grafted or adsorbed protein) at the desired flow rate between 5 and 100 μL/min. Following the injection, the surface was regenerated either by injection of HBS Buffer without calcium (in the case of experiments with the MARCO receptor) or by injections of a solution of 10 mM glycine pH 2.5 + 0.1% Tween 20. In the case of a single cycle kinetics experiment, the analyte was injected at increasing concentrations without regeneration, leaving 300 s between each injection in order to allow for the dissociation kinetics to be studied, followed by subsequent surface regeneration and a repeat of the experiment. The resulting data were analyzed by first subtracting blank injections of running buffer only to account for injection artifacts in the sensorgrams, followed by subtraction of the data obtained on control channel B from channel A to minimize the contribution of non-specific surface interactions and potential buffer effects.

## Supporting Information Available

Information related to ApoE-NT and ApoE-CT protein fragments, lipopolysaccharide assay, adsorption isotherms of ApoE fragments on SiO_2_ NPs, grafting reproducibility studies, electron microscopy characterization of SiO_2_ NPs, characterization of NPs related to Figures 1–3, estimation of the average number of proteins per NP, QCM surface preparation, estimation of immobilized receptor density, injection-regeneration cycles of the immobilized receptor surface, MARCO receptor activity verification with unfolded bovine serum albumin, kinetic analysis model details, and fluorescence emission spectra of ApoE fragments (PDF)

## Abbreviations

ApoE: apolipoprotein E
ApoE R2: apolipoprotein E receptor 2
CT: C-terminus
NT: N-terminus
LDLR: low-density lipoprotein receptor
MARCO: macrophage receptor with a collagenous structure
MPS: mononuclear phagocyte system
NP: nanoparticle
QCM: quartz crystal microbalance
