# Supplementary material for: Quartz Crystal Microbalance Method to Measure Nanoparticle–Receptor Interactions and Evaluate Nanoparticle Design Efficiency
Source: JACS Au. 2023 May 12;3(6):1623–33. doi: 10.1021/jacsau.3c00084 (PMC10301671; doi:10.1021/jacsau.3c00084)
Supplement: Supplementary file 1 — au3c00084_si_001.pdf [file au3c00084_si_001.pdf]

Information related to ApoE-NT and ApoE-CT Protein Fragments:

Alignment of the sequences of the native ApoE3, the fragments with a cysteine in N-term or C-term (ApoE-NT and ApoE-CT respectively). The position of the mutation of the native cysteine for an alanine is indicated in green. The added cysteine in the expressed fragment is indicated in blue. The expressed fragments are also composed of a poly histidine, used for the affinity purification with Ni-NTA columns.

```
Native ApoE3  MKVLWAALLVTFLAGCQAKVEQAVETEPEPELRQQTEWQSGQRWELALGRFWDYLRWVQT 60
ApoE-NT       ---MCGGHHHHHHGGSGGKVEQAVETEPEPELRQQTEWQSGQRWELALGRFWDYLRWVQT 57
ApoE-CT       -----------------MKVEQAVETEPEPELRQQTEWQSGQRWELALGRFWDYLRWVQT 43
                                ******************************************

Native ApoE3  LSEQVQEELLSSQVTQELRALMDETMKELKAYKSELEEQLTPVAEETRARLSKELQAAQA 120
ApoE-NT       LSEQVQEELLSSQVTQELRALMDETMKELKAYKSELEEQLTPVAEETRARLSKELQAAQA 117
ApoE-CT       LSEQVQEELLSSQVTQELRALMDETMKELKAYKSELEEQLTPVAEETRARLSKELQAAQA 103
              ************************************************************

Native ApoE3  RLGADMEDVCGRLVQYRGEVQAMLGQSTEELRVRLASHLRKLRKRLLRDADDLQKRLAVY 180
ApoE-NT       RLGADMEDVAGRLVQYRGEVQAMLGQSTEELRVRLASHLRKLRKRLLRDADDLQKRLAVY 177
ApoE-CT       RLGADMEDVAGRLVQYRGEVQAMLGQSTEELRVRLASHLRKLRKRLLRDADDLQKRLAVY 163
              *********.**************************************************

Native ApoE3  QAGAREGAERGLSAIRERLGPLVEQGRVRAATVGSLAGQPLQERAQAWGERLRARMEEMG 240
ApoE-NT       QAGAREGAERGLSAIRERLGP--------------------------------------- 198
ApoE-CT       QAGAREGAERGLSAIRERLGPGGSGGHHHHHHGGC------------------------- 198
              *********************

Native ApoE3  SRTRDRLDEVKEQVAEVRAKLEEQAQQIRLQAEAFQARLKSWFEPLVEDMQRQWAGLVEK 300
ApoE-NT       ------------------------------------------------------------
ApoE-CT       ------------------------------------------------------------


Native ApoE3  VQAAVGTSAAPVPSDNH 317
ApoE-NT       -----------------
ApoE-CT       -----------------
```

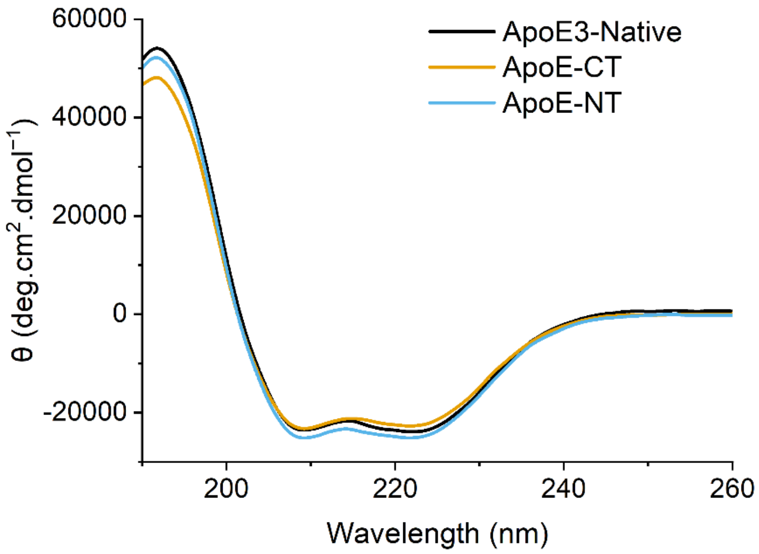

Figure S1. Circular dichroism of the native ApoE3 as well as the 2 expressed ApoE fragments. The expressed fragment have a similar ellipsometry profile compared to the native ApoE, dominated by alpha helix which is in agreement with the protein structure and the literature.[1] This indicates that the secondary structure of the two ApoE fragments is comparable to that of the native ApoE.

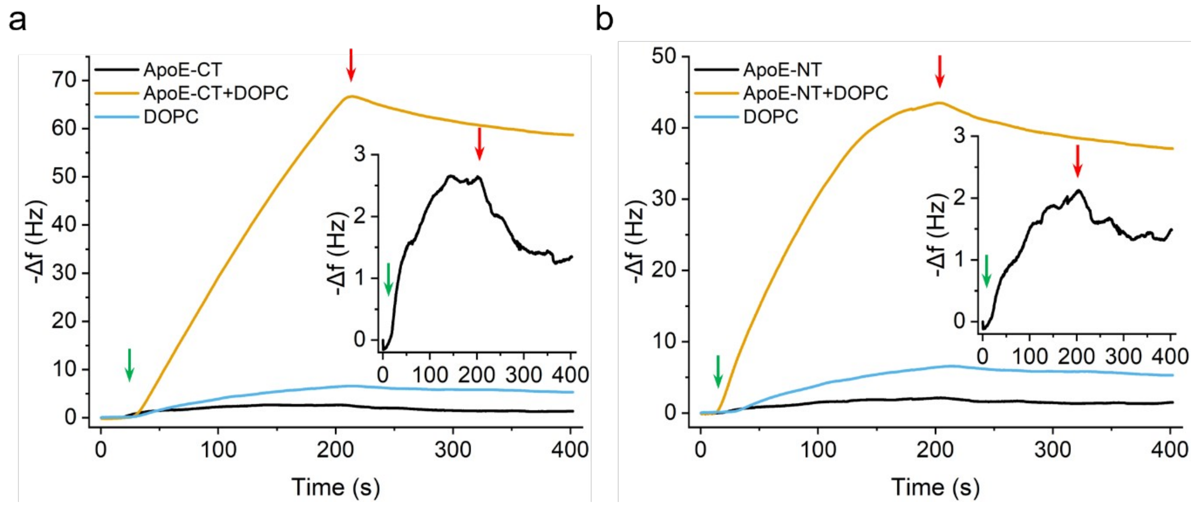

Figure S2. Interaction of ApoE fragments (ApoE-CT in (a) and ApoE-NT in (b)) with ApoER2 in the presence and absence of free DOPC. Prior to the experiment the ApoE fragments were incubated with DOPC small unilamellar vesicles (SUV) in the mass ratio lipid:protein 3.75:1. The inset shows the very weak interaction observed for free ApoE in the absence of DOPC. Injections were made at a protein concentration of 10 µg/mL and at 37.5 µg/mL for the DOPC SUV control.

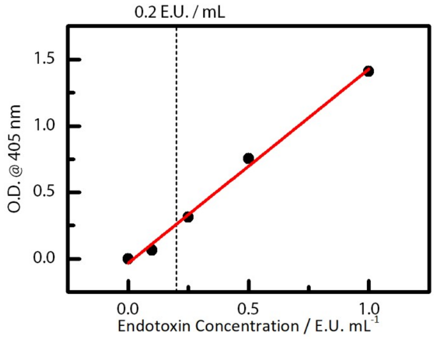

Figure S3: Sample calibration curve obtained from chromogenic assay (Pierce™ LAL Chromogenic Endotoxin Quantitation Kit used in quantification of LPS levels of ApoE-NT and ApoE-CT fragments produced using ClearColi™. The dashed line at 0.2 EU / mL indicates the threshold applied in this work for protein batches to be considered endotoxin free; all ApoE fragments described herein were found to meet this threshold.

Supplementary experimental data related to the functionalisation of the silica nanoparticles with ApoE fragments:

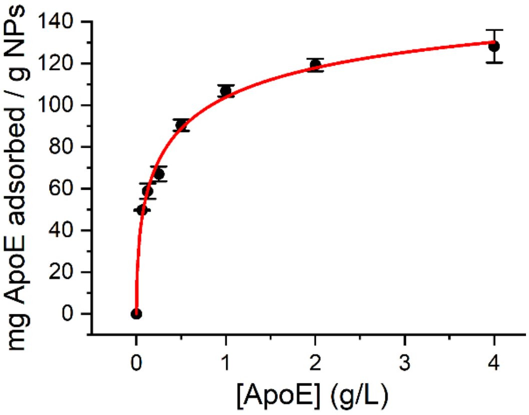

Figure S4. Adsorption isotherm of ApoE fragments on bare SiO_2 NPs at a concentration of 1 g/L. The saturation isotherm corresponds to *ca.* 3000 proteins/NP of 100 nm. Protein concentrations were determined via BCA assay while NP concentration was determined via FitC fluorescence against a calibration line of bare SiO_2 NPs. Values reported are the average of 3 separate measurements. The data were fitted with the Hill equation (line in red). The Hill coefficient obtained from this fit is inferior to 1 and is in agreement with the presence of steric hindrance from the proteins already adsorbed preventing the adsorption of free proteins. The corona formation in this work (NP-corona) was carried out at a concentration of 1g/L because at this point saturation is almost reached while reducing the amount of protein needed.

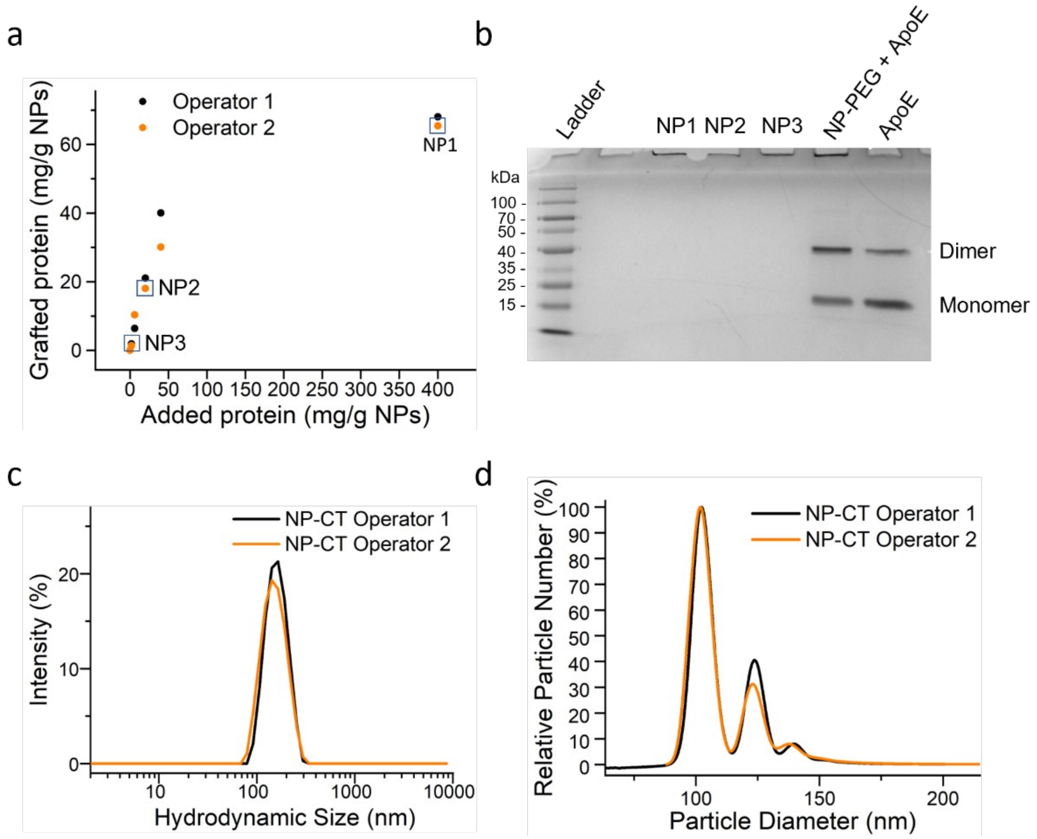

Figure S5: Evaluation of the reproducibility of the grafting of ApoE fragments on silica nanoparticles. a) Estimate of mass of grafted protein obtained per g of NPs as carried out by two independent operators. The mass of protein was estimated via BCA assay carried out according to the manufacturer's protocol, whilst the particle mass was measured by fluorescence against a standard curve of untreated NP-PEG. b) SDS-PAGE (10%) of functionalised ApoE, as indicated in (a). NPs were loaded in each well to have a total of 1 µg protein/well using estimated protein amounts from BCA assays. The absence of a band in the gel indicates that the proteins are covalently attached to the NP surface. A control experiment consisting of blocked NP-PEG incubated with Apo-E under identical conditions to those used in grafting was included, showing that proteins are not simply physisorbed at the particle surface under grafting conditions. The gel was fixed with methanol/aqueous acetic acid and stained via Coomassie staining for 1h before destaining overnight and imaging using a GelDoc® imaging station. c) DLS distribution and d) DCS distribution of the NPs after grafting of the ApoE fragment. These results indicates that the grafting protocol used is reproducible.

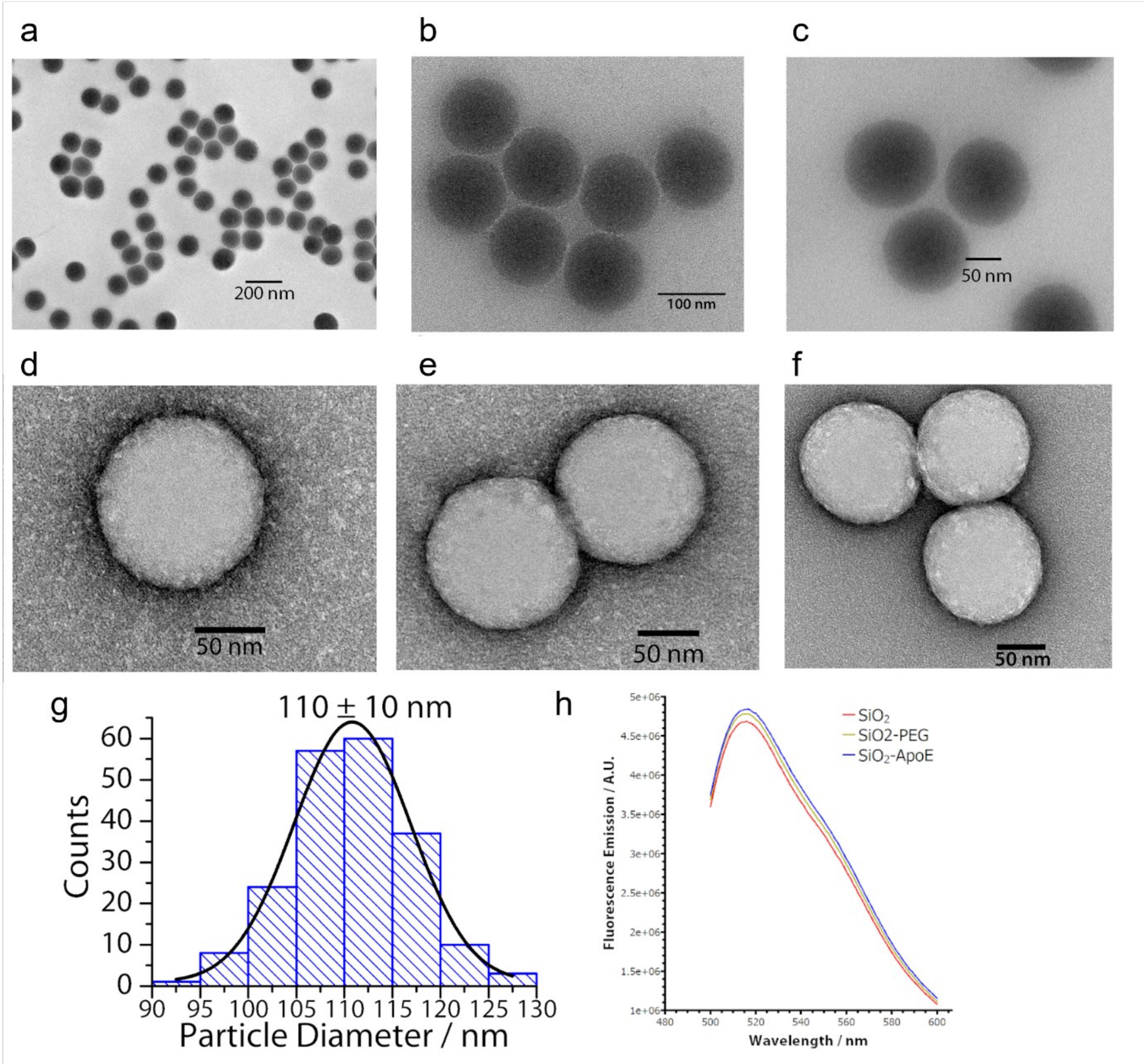

Figure S6: TEM images of a) bare fluorescent SiO_2 NPs, b) NP-PEG and c) NP-CT constructs. d)-f) negative staining of corresponding particles in a)-c), showing the presence of an organic coating for NP-CT that can be attributed to the presence of the protein. Particle size distribution in e) was obtained by counting at least 100 particles across different positions on the TEM grid using ImageJ© software. f) Fluorescence emission of SiO_2, NP-PEG and NP-CT showing that the particles fluoresce due to the presence of FitC dye, and this fluorescence is unaffected by PEGylation and ApoE grafting steps.

a

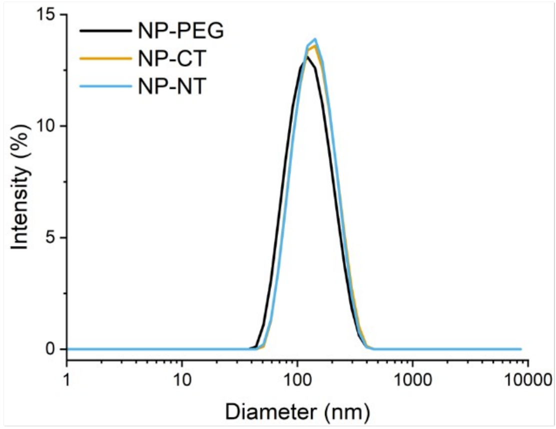

b

Sample	Zav (nm)	PDI
NP-PEG	111 ± 1	0.19 ± 0.01
NP-CT	123 ± 1	0.18 ± 0.01
NP-NT	122 ± 1	0.18 ± 0.01

c

d

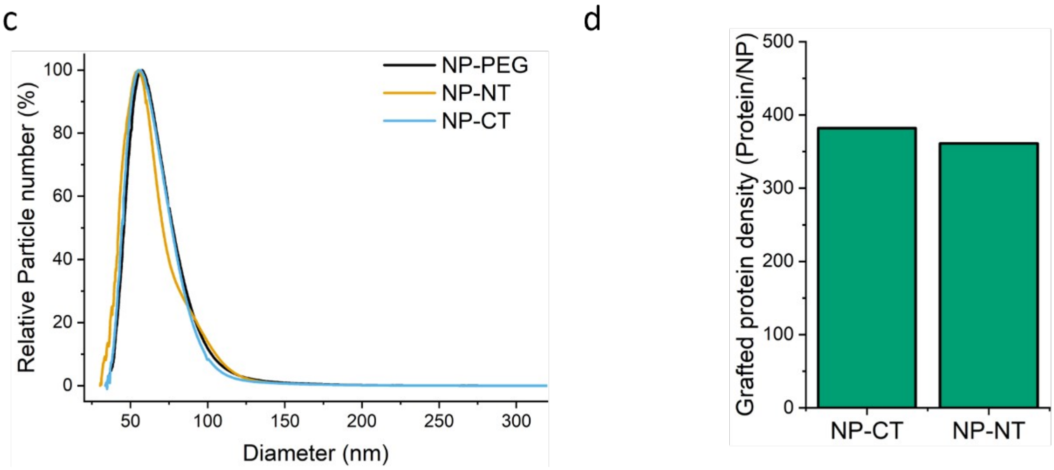

Figure S7: Characterisation of the nanoparticles related to Figure 1. a) and b) DLS characterisation of the NPs dispersed in HBS. c) DCS distribution of the NPs dispersed in PBS. Despite the presence of a secondary peak attributed to NPs dimers, the DCS shows that the NPs do not forms uncontrolled aggregates. The insert show a shift of the main peak toward the smaller size with the increase of the protein grafting density, which is attributed to the reduction of the overall density of the particles (protein layer being less dense that the silica). d) Estimate of the grafted protein density (in number of proteins/NP) according to the BCA assay.

a

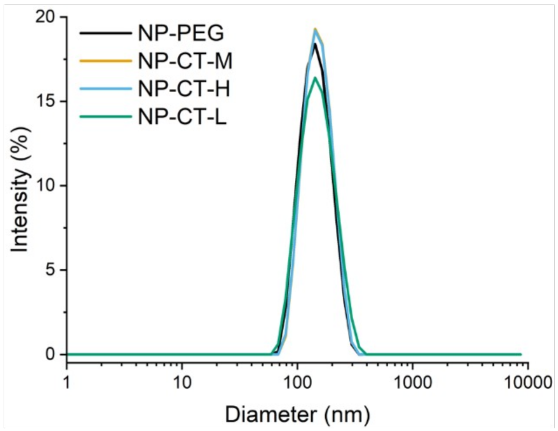

b

Sample	Zav (nm)	PDI
NP-PEG	137 ± 1	0.07 ± 0.01
NP-CT-H	143 ± 3	0.06 ± 0.03
NP-CT-M	144 ± 1	0.06 ± 0.02
NP-CT-L	141 ± 3	0.14 ± 0.03

c d

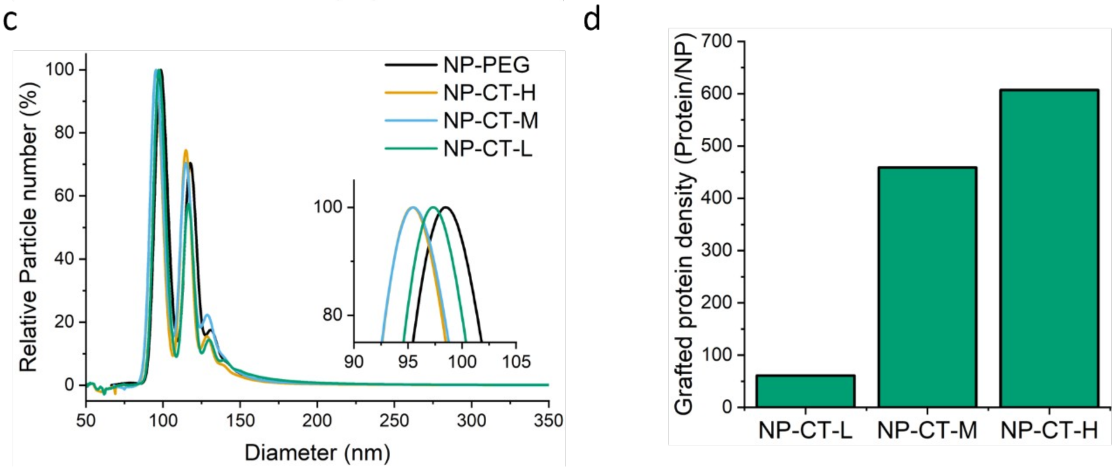

Figure S8: Characterisation of the nanoparticles related to Figure 2. a) and b) DLS characterisation of the NPs dispersed in HBS. c) DCS distribution of the NPs dispersed in PBS. Despite the presence of a secondary peak attributed to NPs dimers, the DCS shows that the NPs do not form uncontrolled aggregates. The insert highlights the shift of the main peak towards smaller sizes with increasing protein grafting densities, which is attributed to the reduction of the overall density of the particles (protein layer being less dense that the silica). d) Estimate of the grafted protein density (in Protein/NP) according to the BCA assay.

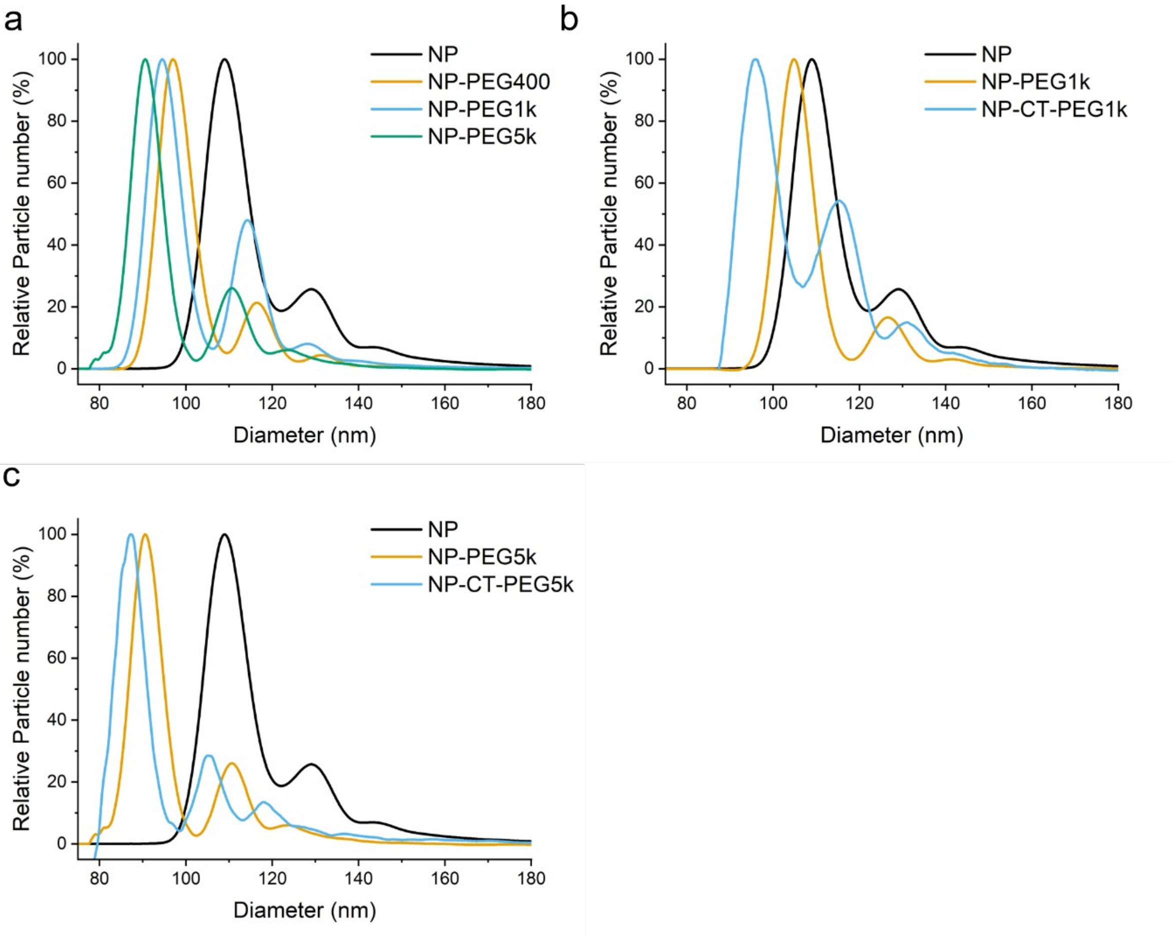

Figure S9: DCS size characterisation of silica NPs with PEG linkers with varying molar mass (400, 1000, 5000 g/mol) related to Figure 3. a) Comparison of unmodified NP with each NP-PEG prepared at the same graft density (20 PEG molecules per nm^2 of surface area). A greater downward shift is observed in the DCS spectra as the molar mass of the PEG increases, consistent with the presence of a thicker PEG layer. b) and c) show size distributions of the same NP-PEG batches after ApoE grafting, confirming that protein grafting induces a further downward shift in apparent size.

Estimation of the average number of grafted proteins per nanoparticles:

$$Protein\ per\ NP = \frac{Protein\ grafting\ density}{1000 \times MW_{Protein}} \times N_A \times \frac{4}{3}\pi . R^3 . d_{SiO2} \quad \text{Equation 1}$$

With *Protein grafting density* expressed in mg of protein per g of silica (measured by BCA), the molecular weight of the protein $MW_{Protein}$ equal to 23000 g/mol, the Avogadro constant N_A (in mol^{-1}), the radius of the nanoparticles R expressed in cm and the density of the colloidal silica d_{SiO2} equal to 2 g/cm^3.

Supplementary QCM Experimental Data

Receptor Immobilisation and QCM Surface Preparation

As described in the experimental section, Attana LNB Carboxyl® QCM chips were prepared for receptor interaction studies by immobilisation of the receptor at the desired amount (2, 20 and 200 hz for low, medium and high receptor densities, respectively). A typical immobilisation profile is shown in Figure S10 below. Both QCM chips (experiment channel A and control channel B) are activated with EDC/NHS to activate carboxyl groups towards reaction with amines, followed by injection of ApoER2 only on channel A until the desired frequency shift is attained. Afterwards two injections of BSA (50 mg/L) are used to block the surfaces of both chips, followed by an injection of ethanolamine (1M, pH 8.5) to block any unreacted NHS esters on the surface.

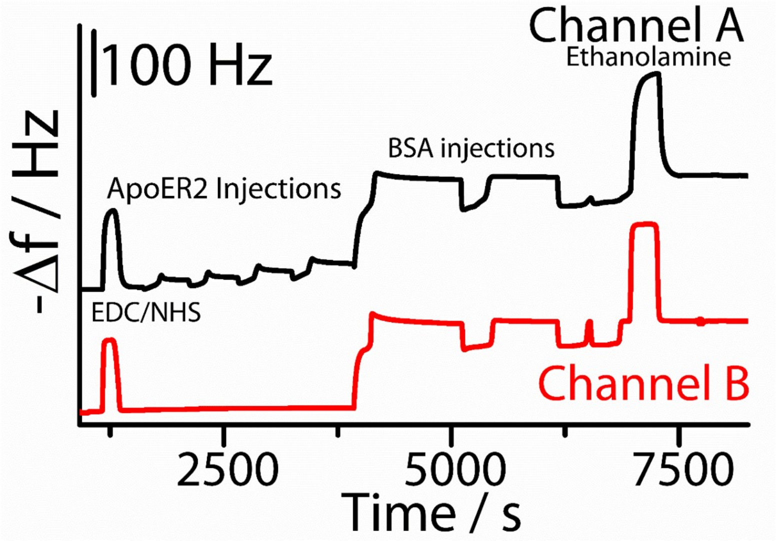

Figure S10. Typical receptor immobilisation and BSA blocking of QCM chips used in experiments detailed in the main text. Prior to the start of experiments, the chips are pre-equilibrated until a drift of no more than 0.5 Hz/min is observed.

Estimation of the immobilised receptor density on QCM surface.

According to the manufacturer specifications, the LNB-Carboxyl® QCM chips purchased from Attana possess an active gold surface with an area of 0.2 cm^2 and Sauerbrey coefficient (C) of 4.4 ng $cm^{-2}Hz^{-1}$. In the case of a rigid layer, by applying the Sauerbrey relation for the immobilised receptor layer (Equation 2) it is possible to estimate the apparent mass of receptor immobilised at the surface:

$$\Delta m = -C \Delta f \quad \text{(Equation 2)}$$

The calculated mass includes also the contribution of coupled water, leading to the overestimation of the receptor density.[2] However since the high, medium and low regimes of receptor immobilisation

differ by an order of magnitude from one another, the Sauerbrey relation is still useful as a relative comparison of immobilised receptor density between surfaces.

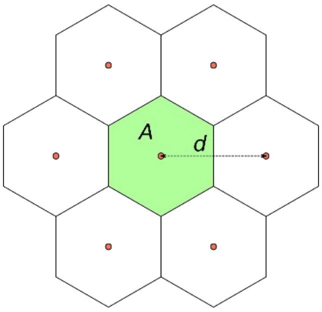

Figure S11: Representation of the receptor (orange dots) distribution on the surface and their associated surface *A*.

Considering that each receptor occupies a hexagonal area surrounded by neighbour receptors occupying an equivalent space (see Figure S11), it is possible to estimate the average distance, *d*, between the receptors:

$$d = 2 \times \sqrt{\frac{A}{6}\sqrt{3}} \quad \text{(Equation 3)}$$

The average distance between the receptor barycentre calculated for the high receptor density corresponds to about 4.4 nm. The fragment of ApoER2 purchased has a calculated mass of 87.5 kDa. If this protein was a sphere with a density of 1.35 g/cm^3 (corresponding to an average globular protein density), the receptor would have diameter of 6 nm. This value is close to the calculated distance between the receptor barycentre, indicating that the surface with the high receptor density is saturated in receptor. The fact that the distance between the receptors is smaller than the calculated size of the receptor can be attributed to the contribution of the coupled water leading to an overestimation of the receptor density, and also to the fact that the receptor is not a perfect sphere.

Table S1. Relationship between estimated receptor density and frequency shift of QCM. Estimated number of receptors per nm^2 of the QCM surface and corresponding average separation between receptors

Receptor Density	Δf QCM (Hz)	Δ*m* (ng/cm^2)	Estimated surface/ Receptors (nm^2)	Average Receptor Separation (nm)
High	180	790	18	~4.6
Medium	20	88	170	~14
Low	2	8.8	1700	~44

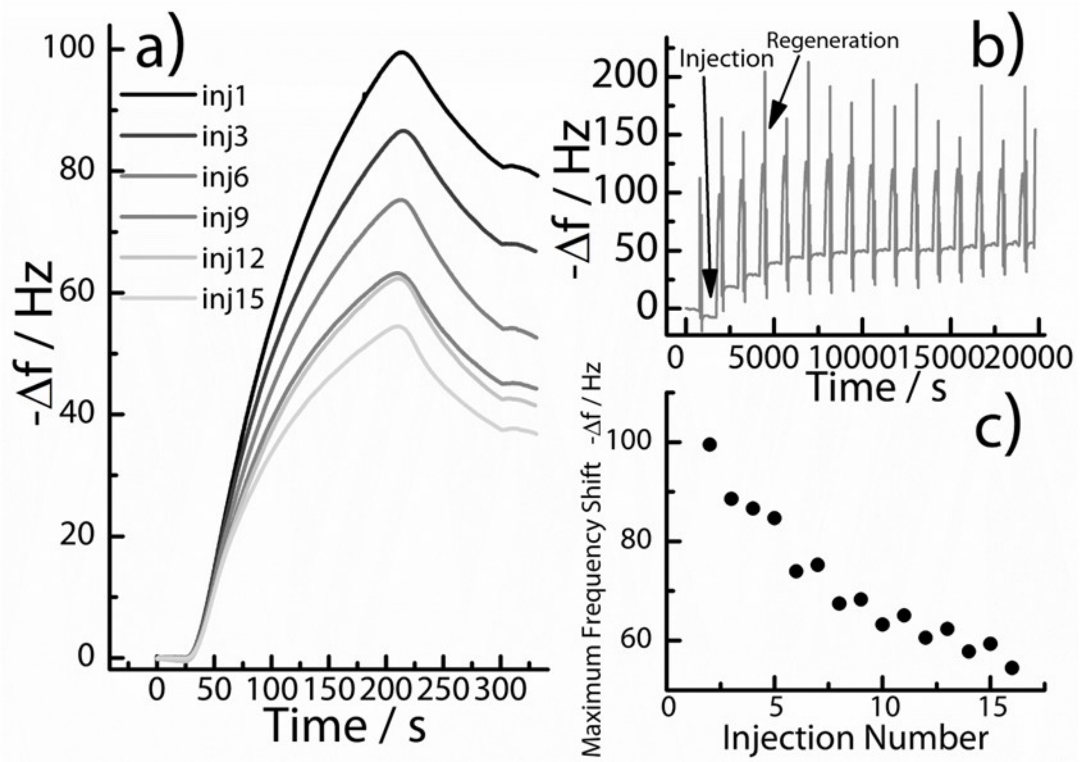

Figure S12: a) Interaction curves of NP-CT (10 µg/mL) on an ApoER2 surface after successive cycles of injection and regeneration with glycine 10 mM pH 2.5 + 0.1 % TWEEN®20. The curves are obtained from raw frequency data (see part b), right) after subtraction of frequency shift of a BSA modified control surface (data not shown) as described in the experimental section. b) raw frequency shift data over time of full injection – regeneration experiment; arrows indicate the NP-CT injection and regeneration. The experiment was repeated 14 times with a period of 10 minutes between each injection. c) plot of maximum frequency shift obtained in part a versus the number of injection cycles.

Figure S12 shows interaction of NP-CT constructs with a single ApoER2 surface following multiple injection-regeneration cycles. The receptor surface retains approximately 90% of its binding capacity after a single regeneration, however, the damage due the regeneration is too significant to regenerate the surface between different concentrations of NPs as used in standard interaction studies. For this reason, we chose to study the binding using a kinetic titration experiment, also known as single cycle kinetics. This approach is well established as an alternative to the standard method and yields identical kinetic information[3]. Moreover, when designing the experiments, NPs with the weakest interaction were placed first in the sequence of injection (surface with the lowest amount of inactive receptors), and the entire experiment sequence was duplicated in order to account for error due to surface damage.

Verification of activity of MARCO receptor surface using unfolded Bovine Serum Albumin

To verify that MARCO surfaces used in interaction studies were functional, we prepared solutions of unfolded endotoxin-free BSA by two successive heat treatments (80 ºC, 2 min) followed by vortexing (1 min). After cooling to room temperature, large aggregates were removed by centrifugation (20000×g, 10 min) and the solution was injected into the QCM at a concentration of 10 mg/L. Figure S13 shows a typical association-dissociation curve for unfolded BSA under these conditions on the MARCO surface employed for tests of NP-CT recognition (Figure 4 in the main text).

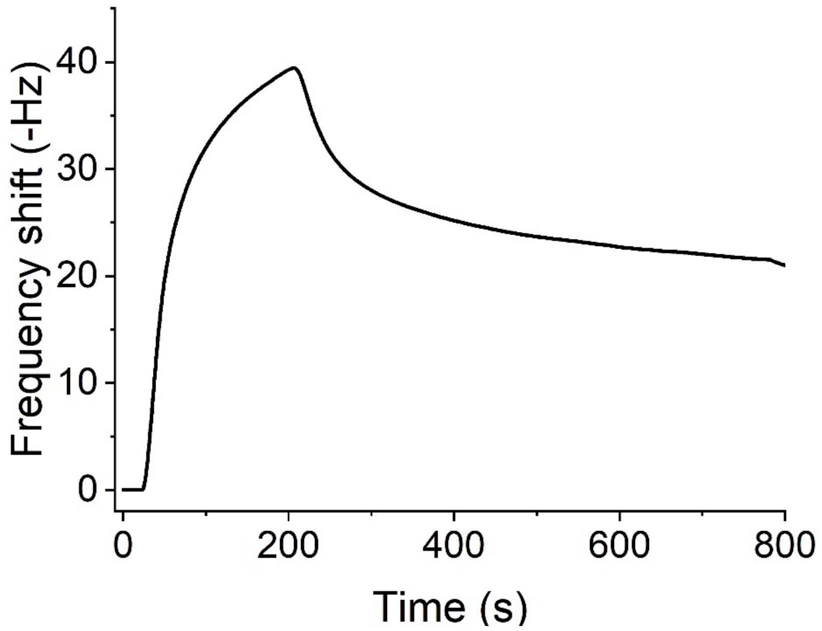

Figure S13. Interaction of unfolded, Endotoxin-free BSA (10 mg/L) with a MARCO receptor surface, showing the functionality of the immobilised receptor.

Kinetic analysis model:

Association-dissociation curves were recorded on the Attana Cell™ 200 by monitoring and recording the QCM frequency over time with proprietary Attester® software. This was carried out on both receptor and control (BSA) chip surfaces (channel 'A' and 'B') simultaneously, as described in the main text. The resulting kinetic curves were corrected for any non-specific interactions by subtraction of data of channel B from channel A prior to exporting.

The binding kinetics were determined using ClampXP™ software, which may be used to fit QCM data of this type through a model-building approach. The most common model used in protein-receptor interactions is the simple 1:1 model:

$$A + B \underset{k_{off}}{\overset{k_{on}}{\rightleftharpoons}} AB \quad \text{Equation 4}$$

Here A describes the analyte (protein), B the immobilised receptor and AB the resulting protein-receptor complex. Binding is reversible and occurs at rates described by the association (k_{on}) and dissociation (k_{off}) rate constants. The measured QCM frequency shift R (Hz) is directly proportional to the density of AB sites.

The association and dissociation obtained with the nanoparticles clearly indicated that the 1:1 model is not appropriate to describe this particular system. Instead we consider a model with 2 different binding sites on the surface, a monovalent and a multivalent binding site, as described in the main text and illustrated in Figure 3 e. As an approximation we consider that the multivalent and monovalent site present the same apparent association constant, because even in the case of a multivalent interaction the first interaction remains monovalent, but then the multivalent site is associated to a smaller apparent dissociation constant because the dissociation of the NPs from the surface requires the dissociation of all the different NP-receptor pairs. The model can then be presented as:

$$\begin{cases} A + B_{mono} \underset{k_{off(mono)}}{\overset{k_{on}}{\rightleftharpoons}} AB_{mono} \\ A + B_{Multi} \underset{k_{off(multi)}}{\overset{k_{on}}{\rightleftharpoons}} AB_{multi} \end{cases} \qquad \text{Equation 5}$$

Table S2. Fit parameters using a heterogeneous surface model as described above for the NP-CT-600 – on low and medium ApoER2 density (from Figure 3)

Protein graft Density	k_{on} (M^{-1} s^{-1})	$k_{off(mono)}$ (s^{-1})	$k_{off(multi)}$ (s^{-1})	B_{multi}/ B_{mom}	$K_{d(mono)}$ (M^{-1})	$K_{d(multi)}$ (M^{-1})
Low ApoER2 density	$2.7.10^{7}$	7.10^{-3}	1.10^{-3}	0.3	3.10^{-10}	4.10^{-11}
Medium ApoER2 density	1.10^{7}	5.10^{-3}	1.10^{-3}	1.6	5.10^{-10}	1.10^{-10}

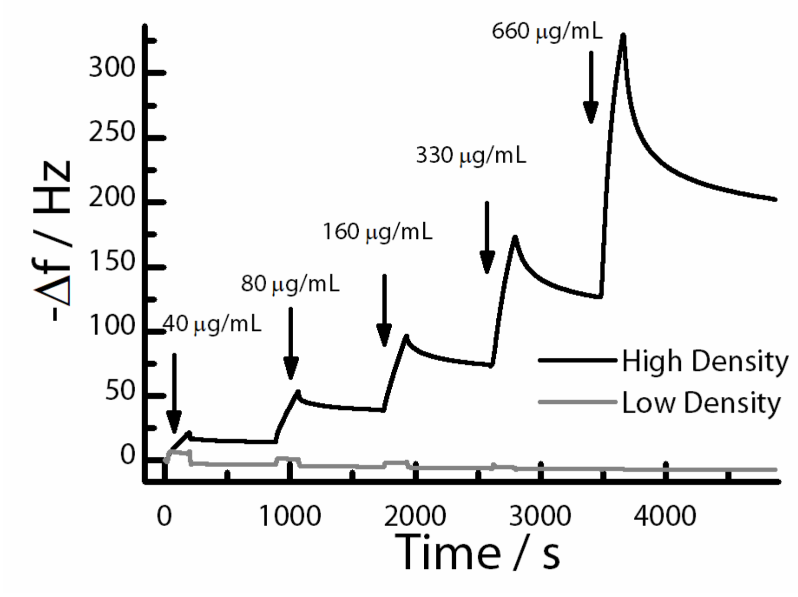

Figure S14. Interaction of NP-CT constructs prepared using PEG-1000 linkers with a high receptor density of ApoER2. As for NP-PEG-8 bionanoparticles, at high protein density (~600 proteins/NP) the particles are recognised by ApoER2, while at low (60 proteins/NP) immobilisation densities, no interaction is observed at any injection concentration.

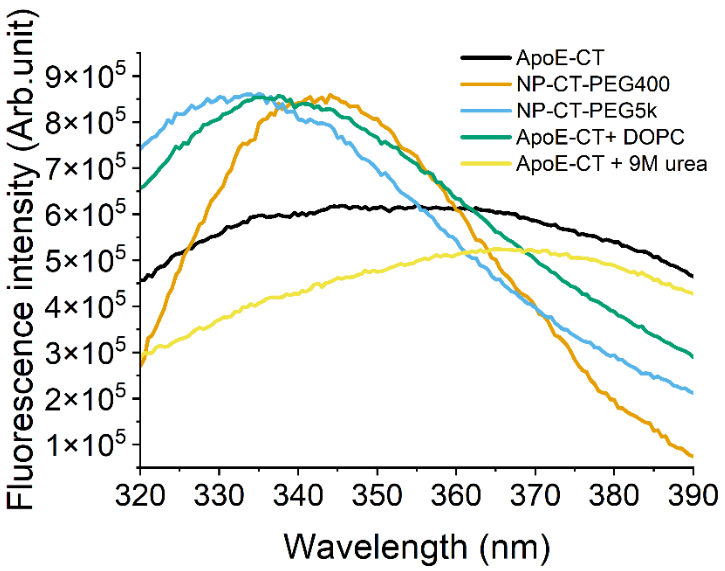

Figure S15. Fluorescence emission spectra of solutions of ApoE / grafted ApoE bionanoparticles in the region from 320-390 nm corresponding to tryptophan residues. Proteins (10 µg/mL) were incubated overnight in solutions of DOPC (to allow for lipid binding) or urea 8M (to induce protein unfolding) in order to assess the effect on protein conformation. Both lipid binding and PEGylation appear to result in similar conformations, distinct from native or unfolded ApoE fragments.

References:

1. Aggerbeck, L. P.; Wetterau, J. R.; Weisgraber, K. H.; Wu, C.-S. C.; Lindgren, F. T., Human Apolipoprotein E3 in Aqueous Solution. II. Properties Of The Amino- And Carboxyl- Terminal Domains. **1988,** *263* (13), 6249-6258.
2. Höök, F.; Kasemo, B.; Nylander, T.; Fant, C.; Sott, K.; Elwing, H., Variations in Coupled Water, Viscoelastic Properties, and Film Thickness of a Mefp-1 Protein Film during Adsorption and Cross-Linking: A Quartz Crystal Microbalance with Dissipation Monitoring, Ellipsometry, and Surface Plasmon Resonance Study. *Anal. Chem.* **2001,** *73* (24), 5796-5804.
3. Karlsson, R.; Katsamba, P. S.; Nordin, H.; Pol, E.; Myszka, D. G., Analyzing a kinetic titration series using affinity biosensors. *Analytical Biochemistry* **2006,** *349* (1), 136-147.
